# Post-Exercise Sweat Loss Estimation Accuracy of Athletes and Physically Active Adults: A Review

**DOI:** 10.3390/sports8080113

**Published:** 2020-08-11

**Authors:** Eric O’Neal, Tara Boy, Brett Davis, Kelly Pritchett, Robert Pritchett, Svetlana Nepocatych, Katherine Black

**Affiliations:** 1 Department of Kinesiology, University of North Alabama, Florence, AL 35633, USA; tgoldman@una.edu; 2 Department of Kinesiology, Auburn University at Montgomery, Montgomery, AL 36166, USA; bdavis56@aum.edu; 3 Department of Health Sciences, Central Washington University, Ellensburg, WA 98926, USA; Kelly.Pritchett@cwu.edu (K.P.); Robert.Pritchett@cwu.edu (R.P.); 4 Department of Exercise Science, Elon University, Elon, NC 27244, USA; snepocatych@elon.edu; 5 Department of Human Nutrition, University of Otago, Dunedin 9054, New Zealand; katherine.black@otago.ac.nz

**Keywords:** hydration strategies, team sports, endurance, fluid balance, sweat

## Abstract

The main purposes of this review were to provide a qualitative description of nine investigations in which sweat losses were estimated by participants following exercise and to perform a quantitative analysis of the collective data. Unique estimations (*n* = 297) were made by 127 men and 116 women after a variety of exercise modalities in moderate to hot environmental conditions. Actual sweat loss exceeded estimated sweat loss (*p* < 0.001) for women (1.072 ± 0.473 vs. 0.481 ± 0.372 L), men (1.778 ± 0.907 vs. 0.908 ± 0.666 L) and when all data were combined (1.428 ± 0.806 vs. 0.697 ± 0.581 L), respectively. However, estimation accuracy did not differ between women (55.2 ± 51.5%) and men (62.4 ± 54.5%). Underestimation of 50% or more of sweat losses were exhibited in 168 (54%) of estimation scenarios with heavier sweaters displaying a higher prevalence and trend of greater underestimations in general. Most modern guidelines for fluid intake during and between training bouts are based on approximate sweat loss estimation knowledge. These guidelines will likely have minimal efficacy if greater awareness of how to determine sweat losses and accurate recognition of sweat losses is not increased by coaches and athletes.

## 1. Introduction

Over the last two and a half decades, multiple scientific cohorts and conclaves have devoted extensive efforts to provide physically active individuals with robust guidelines for fluid intake strategies during and in the times between training bouts [1,2,3,4,5,6,7]. While some viewpoints have contended that thirst is an adequate indicator and stimulus for proper fluid consumption [6,8], most other guidelines include detailed recommendations for ideal fluid intake practices based on approximate estimates of sweat losses that will be incurred [1,3,4,7]. There is no lack of original research concerning hydration and physical activity, but one article in particular, Passe et al. [9], piqued the interest of the current authors in regards to whether the more nuanced guidelines have pragmatic value in real world practice. 

Passe et al. [9] reported that during a 16-km run trained runners severely under replaced sweat losses with fluid intake (replacing 30.5% ± 18.1% of sweat loss) in comparison to formal contemporary hydration recommendations of that period suggesting fluid intake match sweat losses [1]. This occurred despite fluid intake opportunities that were designed to be as accommodating as possible. The recommendation of complete fluid replacement during exercise [1] that the investigators used to interpret the adequateness of fluid consumption has since been deemed excessive and that body mass loss during exercise should occur [4]. However, to our knowledge Passe et al. [9] also presented the first evidence that reported athletes’ perceptions of sweat losses, with an average underestimation of sweat loss volume of ~43%. Beverage intake adequateness changes drastically if replacement volume is viewed from the standpoint of the runners’ perceived sweat loss volume. This finding is revelatory but almost universally disregarded when discussing the efficacy of nearly all contemporary, formal hydration guidelines that base fluid replacement recommendations on individual sweat loss volume.

Since the seminal article that reported sweat loss estimation accuracy, multiple attempts to document athletes’ perception of sweat loss volume have been undertaken in the current authors’ respective laboratories. However, there has been no formal review of these sweat loss estimation investigations. The two primary purposes of this review will be to provide a single, concise literature overview source of sweat loss estimation investigations and combine individual data from these studies for a comprehensive descriptive analysis of athletes’ sweat loss estimation accuracy in field and laboratory settings. A tertiary aim was to offer some brief, real world application considerations based on these findings for coaches, athletes and sport medicine staff. 

## 2. Methods

The lead author initiated communications with all authors of investigations that had cited his original sweat loss estimation study [10] and collected original sweat loss estimation data in their respective publications. Although not a formal systematic review, each author then completed their own individual search of literature that cited their original sweat loss estimation studies. No additional published articles were discovered. Data from nine published investigations [10,11,12,13,14,15,16,17,18] in which sweat loss was estimated were shared by current authors through personal communication. A new spreadsheet database was created for comprehensive analysis. Data included a description of participants (age, sex and training background) and physical activity conditions (exercise modality, environmental conditions and duration of exercise). Additionally, pre-activity body mass, post activity body mass, fluid intake and void output (if applicable) were used to calculate actual sweat loss, post-exercise estimated sweat loss volume and sweat loss as a percent of body mass. Sweat loss was calculated as change from pre- to post-exercise body mass with adjustment for fluid intake or urine production and under the assumption that 1 kg of body mass change was equal to 1 L of sweat loss. The authors recognize that sweat loss is more complex than this simple model due to the presence and density of electrolytes and metabolites found in sweat [19] and respiratory water and gas exchanges related to metabolic processes [20]. However, due to the variety of exercise modalities and duration of exercise for most of the investigations, the authors have chosen to represent sweat loss volume in liters as equivalent to change in body mass in kilograms. 

Each investigation is summarized in Table 1. Two running studies [11,18] and one basketball study [12] incorporated designs in which sweat loss estimations were made twice by the same participants during separate training sessions. In these studies, participants were not informed of their actual sweat losses until after study completion. Thigpen, Green and O’Neal [12] examined sweat loss estimation in short conditioning practices and longer, in-season practices, and Davis et al. [18] looked at sweat loss estimation during treadmill running under temperate versus hot conditions. Both of these studies subcomponents are presented in separate rows in Table 1 due to the dramatic difference in testing conditions. The findings of Shaver et al. [11] are not presented in two rows due to the consistency in exercise modality, duration and environmental conditions. Muth et al. [16] also incorporated multiple sweat loss estimations within subjects, but participants were informed of their sweat loss volume after the first estimation session. Thus, only first sweat loss estimation accuracy scores are presented in Table 1 for [16], but data has been presented separately for male and female rugby players.

### Statistical Analysis

Following testing for equal variance, independent samples *t* test were used to analyze differences between sexes for age, weight, duration of exercise, sweat loss (absolute and as percent body mass) and sweat loss estimation accuracy. Dependent *t* tests were used to compare absolute and estimated sweat losses within each sex and for all data combined. Three scatterplots were prepared using data from all studies. In the first scatterplot, estimated sweat loss ((estimated sweat loss/actual sweat loss) × 100) is plotted relative to actual sweat loss. The second and third plotted sweat loss estimation accuracy as a percentage against sweat loss as a percent body mass ((sweat loss/body mass) × 100) with markers identifying participants by study or by sex. Additionally, a contingency table was prepared to categorize outcomes from the scatterplots. All data were analyzed and figures were created using Microsoft Excel. Because of the large sample size, an alpha of 0.01 was selected a priori. All data are presented as mean ± SD.

## 3. Results

**Figure 1:**
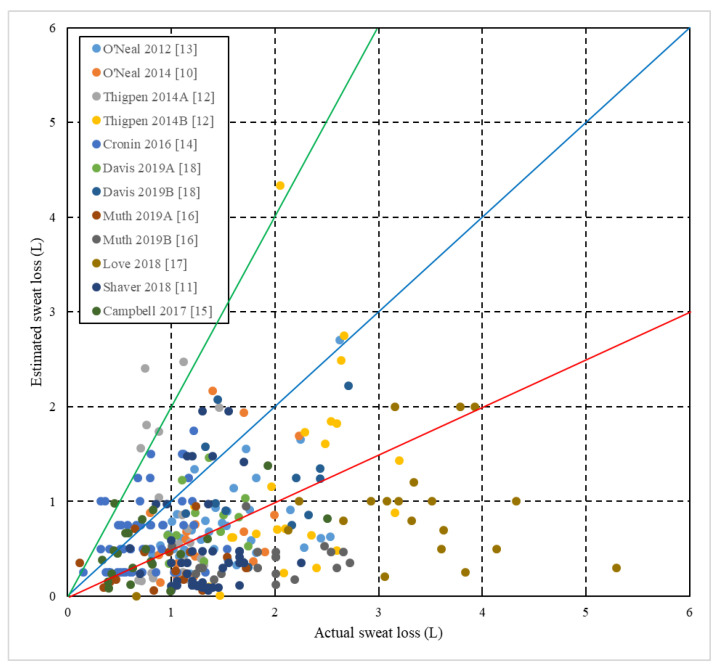
Individual (*n* = 297) sweat loss estimations versus actual sweat losses across all studies. The middle blue line represents 100% accuracy in prediction. Markers below the red line represent 50% or greater underestimation. Markers above the green line represent 50% or greater overestimation. Dashed, vertical and horizontal lines separate absolute and estimated sweat loss volumes into 1 L increments.

**Figure 2:**
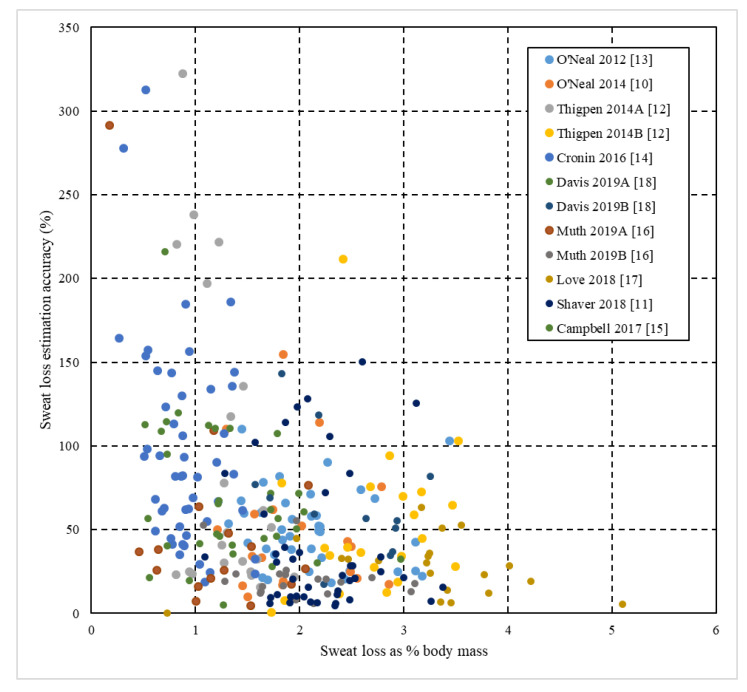
Relationship between sweat loss presented as a percentage of body mass and sweat loss estimation accuracy (%) (*n* = 297) across all studies with coded identification by study.

**Figure 3:**
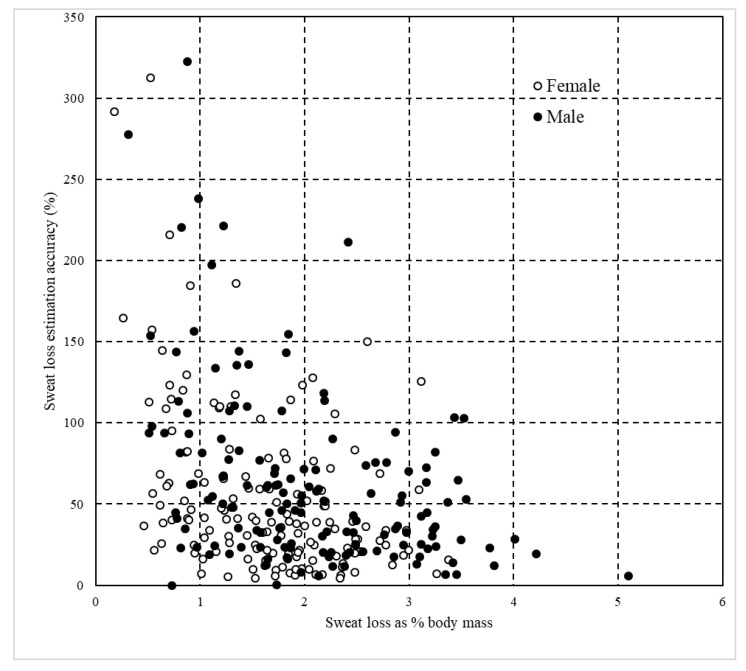
Relationship between sweat loss presented as percentage of body mass and sweat loss estimation accuracy (%) (*n* = 297) across all studies with coded identification by sex.

The nine studies included 243 participants (males = 127; females = 116) and produced 297 unique samples (Table 1). All participants were at least 18 years of age and varied in experience from recreational exercisers to professional athletes (Table 1). Exercise modalities included hot yoga, running, rugby, basketball and CrossFit^™^ training (Table 1). Female particpants exhibited lower (*p* < 0.001) body masses, sweat loss as a percent body mass, absolute sweat losses and sweat loss estimations than male participants despite no difference in exercise duration (Table 2). Actual sweat losses were approximately double the estimated sweat loss volumes and significantly differed for female, male and all participants, but there were no significant differences in the accuracy of predictions by percentage between male and female participants (Table 2). Table 3 and Figure 1, Figure 2 and Figure 3 collectively further highlight the high prevalence of underestimation with participants experiencing greater absolute and relative sweat losses more heavily underestimating sweat loss volume.

## 4. Discussion

The first aim of this paper was to provide a review of the authors’ individual investigations concerning sweat loss estimation accuracy under a variety of exercise modalities and environments in a single source (Table 1). The second goal was to determine if sweat loss estimation trends across multiple conditions were consistent when viewed from a more global perspective. Vast efforts have been made by the scientific community to promote hydration strategies centered on the recognition of individual sweat rates [1,3,4,7]. However, data from the current study indicate athletes and adults engaging in exercise underestimate sweat losses by around 40–50%. Long distance runners are potentially the most likely population of athletes that could benefit from knowing their sweat loss due to the duration and environmental conditions encountered in competition, but survey literature has repeatedly confirmed that runners are unlikely to be knowledgeable about or use their actual sweat rates to determine fluid intake [21,22,23]. In regards to our second objective, cumulative data (Table 2 and Table 3 and Figure 1, Figure 2 and Figure 3) provide overwhelming clarity that the vast majority of athletes and physically active adults underestimate sweat loss in temperate to hot environments regardless of sex or exercise modality.

It is important to recognize a potential overestimation interpretation bias in individual studies (Table 1). The nature of estimating sweat loss dictates significant outliers are only possible in the direction of overestimation which has no ceiling, while underestimation has a floor of 0%. This structure allows outliers in the smaller samples of individual studies to have greater capacity to pull the mean in the overestimation direction. Considerable overestimation was also almost exclusively limited to conditions in which sweat losses were absolutely or relatively low. This trend is exemplified by viewing Figure 2 where 16 of 183 (8.7%) overestimations exceeding 150% of sweat loss were for individuals that lost less than 2% of their body mass. Comparatively, only 2 of 114 (1.8%) participants whose sweat loss exceeded 2% body mass overestimated losses in excess of 150%. The color coding in Figure 1 and Figure 2 identifies that 13 of the 18 overestimations >150% were from conditions in which very high intensity exercise was undertaken during a short activity bout including a CrossFit^™^ workout [14] and a 46 min collegiate men’s basketball team conditioning practice [12]. Combined with less opportunity for convective induced sweat loss evaporation, these outlier overestimations likely explain why these two study conditions present the two highest estimation accuracy or overestimation averages (Table 1).

Fluid intake greatly exceeding sweat losses increases chance of developing hyponatremia and is a critical but possibly overlooked driving force for fluid intake guidelines during exercise being based on individual sweat rate. Although complicated by relative body mass and other factors such as longer average finish times during endurance competition, female endurance athletes are more likely to experience exercise associated hyponatremia [6]. Profiles of women undertaking light to moderate exercise intensity in hot conditions and consuming fluids greatly exceeding sweat loss [24,25] even to the point of symptomatic hyponatremia (defined as a serum sodium <135 mmol·L^−1^) in laboratory settings [26] have been reported. It is plausible that in incidents where females have exhibited higher rates of hyponatremia than male counterparts could partially be attributed to a propensity of greater sweat loss overestimation leading to gratuitous fluid intake. Although absolute and relative sweat losses were lower for female participants (Table 2), only 8 of the 18 overestimations of >150% were made by women (Figure 3). There is no visual trend supporting that significant overestimation of sweat loss is greater in physically active women versus men (Figure 3), indicating that overestimation of sweat loss is not the reason for higher fluid intakes and rates of hyponatremia amongst active females. 

Using education to alter hydration behavior has resulted in equivocal outcomes [27,28,29]. A total of nine runners in O’Neal et al. [10] reported previously measuring change in body mass to determine sweat loss volume before study initiation. However, these participants were no better at estimating their sweat losses after a 1 h run in warm weather than the remaining 30 runners who were unaware this method could be used to determine sweat losses. Runners’ sweat loss estimates also showed no difference before or after the run. Only one investigation incorporated a design in which participants were made aware of sweat losses to determine if they could improve estimation accuracy over time. Muth et al. [16] reported underestimation accuracy improving from 81 ± 13 (practice 1) to 38 ± 30% (practice 3) for male, collegiate rugby player. Female athletes in the same study improved from 64 ± 71 to 43 ± 37% underestimation accuracy from practice 1 to practice 2, but fell back to 60 ± 212% on practice 3. The 3rd highest overestimation presented from any study in Figure 2 came from this female cohort [16], and as described above, a few very high overestimations skewed the capacity for growth in estimation accuracy. It is also important to note that regardless of sex or education on sweat losses, the rugby players still overwhelmingly chose estimations below actual sweat losses. During two separate 15-km runs in similar environments with no feedback on sweat losses during the initial run session, Shaver et al. [11] reported no differences in absolute sweat loss or estimation accuracy between runs. Although not reported in the original paper, the current authors reevaluated the data used in the current study and found a strong relationship between sweat loss estimations for the first and second runs (intraclass correlation = 0.89, *p* < 0.001) further indicating participants’ perceptions of sweat losses were consistent. In the only other repeated measures design study where exercise modality and duration were held constant, sweat loss estimation accuracy by percentage was found to not differ between temperate versus hot environment treadmill runs of 60 min [18].

Despite what is most likely the sample with the greatest resources to nutritional education and the only study with professional athletes, Love et al. [17] found elite rugby players displayed no distinctive edge in estimating sweat losses compared to recreational or collegiate athletes (Table 1, Figure 1 and Figure 2). To the contrary, the average underestimation percentage was higher than all other studies excluding the group of collegiate, male rugby players (Table 1). Further, the magnitude of the difference between measured and estimated sweat loss was not different between those who self-reported using hydration monitoring strategies during and between training sessions and those who did not. No player reported using change in body mass pre- to post-practice or between practices as a hydration monitoring technique, likely explaining the lack of differentiation between groups’ sweat loss estimation accuracy. Urine color was reported to be utilized to determine pre-practice hydration by 78% of players that used a hydration monitoring technique. Urine color can generally be a reliable indicator of significant dehydration in real time during prolonged exercise in the heat [30], but provides no quantitative sweat loss volume information. Urine color and change in body mass from practice to practice are techniques athletes and coaching or sport medicine staff use to assess hydration status in team sports, but the authors are unaware of any literature supporting intentional recognition of sweat rate as a method incorporated for team sport athletes as a common practice.

The final aim of this study was to provide a connection from current findings to real world practice. The current authors suggest the first consideration is determining if sweat loss assessment practices are actually warranted. If so, the next step is to determine if the sweat loss estimation information collected will be used in the context of developing strategies concerned with fluid intake during exercise or between training bouts. In regards to fluid consumption during exercise, sweat rate information is mostly irrelevant for the vast majority of physical activity scenarios. Adults engaging in recreational exercise are highly likely to consume enough fluid during training and experience sweat losses low enough that they are unlikely to experience a loss in body mass exceeding the 2% threshold that is commonly proposed to be associated with performance impairment [14,15,31]. When given free access to beverages, the majority of team sport athletes will also drink ad libitum at a rate sufficient to prevent severe dehydration ad libitum [12,32,33]. Collectively, ad libitum fluid consumption does not appear to degrade performance of endurance athletes training or competing for durations of 60–120 min bouts [34]. However, there are likely many individual endurance athletes that could potentially benefit from adjusting fluid intake strategies based on knowledge of expected sweat losses, particularly those of longer durations [35,36]. Although only anecdotal, following the O’Neal et al. [10] study investigators received unsolicited notifications from multiple participants expressing personal best events in long distance running or triathlon events after altering fluid intake patterns based on recognition of their actual sweat rates. All of these individuals greatly underestimated their sweat losses during the 1-h run in warm environmental conditions of the study. While assessing approximate sweat loss does not require great technical skills and the only equipment needed is a calibrated scale, the current authors contend this process is only necessary for during exercise fluid intake strategy preparation if (1) exercise conditions will incur sweat losses greatly exceeding 2% body mass (e.g., half marathon participation in warm environmental conditions) or (2) to prevent overconsumption of fluids during events that might lead to exercise associated hyponatremia (e.g., a 5 h marathon finish in cool environmental conditions). It is also paramount that individuals recognize their sweat rate can be affected by change in environmental conditions, acclimatization and state of training.

Recognizing sweat loss volume to improve between training bout fluid intake has a more universal appeal. Carrying fluids on oneself and drinking while running can be more difficult than in team sport settings that include regular pauses in activity. Competitive runners and endurance athletes are also apt to train more than once in a 24-h period. For these reasons our laboratories have made multiple efforts to examine ad libitum hydration behavior, fluid dynamics and practical hydration marker validity for ~12 h following runs that produce significant sweat losses [13,18,37,38]. Change in absolute body mass from one training session to the next is commonly promoted in field settings as method to assess hydration status. However, this technique may not be a great indicator of adequate fluid replacement between training bouts. Davis and colleagues [37] reported that trained runners’ body masses differed by only ~0.5 kg the following morning after a 75 min evening run in hot conditions despite metered beverage intake replacing 75% (1637 ± 372 mL) versus 150% (3099 ± 850 mL) of sweat losses throughout the recovery period. The runners’ next morning performance 10-km time trials were impaired by 3% during the 75% versus 150% fluid replacement trials, and pre-run urine specific gravity was 0.012 units higher for 75% fluid replacement [37]. We have recently demonstrated that runners’ ad libitum rehydration efforts following runs are consistent within runners even between temperate and hot training conditions [18], and that high urine specific gravity is a strong indicator of inadequate fluid intake between training sessions separated by ~12 h, particularly if sweat losses exceed 3% body mass [39]. Sports medicine staff for endurance athlete teams might consider periodic pre-practice urine specific gravity assessment to identify athletes that consistently fail to adequately consume fluids between practices. Staff could then move to assess and educate athletes most needing increased recovery fluid intake strategy modifications based on sweat loss volume during training. This approach may also help mitigate the stressors of regularly weighing in for endurance athletes that may suffer from body dysmorphia issues. For the many endurance athletes training without sports medicine staff supervision, the recognition of sweat loss and self-assessment of fluid intake during recovery using metered beverage bottles can help confirm the adequacy of individual between training bout hydration strategies without the need for expensive equipment or technical expertise.

Team sport practice studies described in Table 1 [12,16,17] provide evidence that considerable sweat losses can be expected during formal collegiate or professional team practices. Further inquiry into each of these three studies also reveals vast cases of athletes with high urine specific gravity values associated without inadequate fluid replacement between practices [39]. As also suggested for endurance sport teams, the authors recommend that multiple sport urine specific gravity evaluations can used to expediently determine which team athletes may be in most need of further guidance to improve between training bout fluid intake. Once identified the extra step of determining approximate sweat losses per practice for those athletes indicating most need for intervention can be initiated. Team sport athletes can drink significant volumes of fluid during practice [12,16,17] which may make the sweat loss estimation process a little more complex and highlights the need to selectively incorporate this approach for time and staff efficiency with large teams. Many team sport athletes have individual bottles assigned to drink out of during practices. Thigpen and colleagues [12] asked basketball players in their study to estimate their sweat loss volume by filling the actual model of sport bottles they used during practice and carry with them throughout their day. The authors followed up their investigation with a well-received hydration education forum for the team in which the athletes were given their sweat loss information and the volumes of sweat loss were visually demonstrated using their own sport bottles. Helping athletes visualize their sweat loss volume may be an effective educational approach on hydration.

Sweat loss estimation accuracy was a secondary aim for Passe et al. [9] and almost all of the investigations presented in Table 1. However, together these studies provide significant practical insight into the mindset of the target populations when designing fluid intake guidelines for athletes and physically active individuals. Collective analysis of the nine investigations included in this review provide compelling evidence that physically active adults cannot accurately estimate their sweat losses in ecologically valid scenarios (e.g., team practices, outdoor runs, fitness classes, etc.) if environmental conditions are temperate or hot. The overwhelming error direction is skewed toward underestimation of sweat losses. This tendency is not specific to sex or exercise type. Before sweat loss evaluation protocols are implemented coaches or athletes should take into consideration if their training regimen is expected to produce significant sweat loss or if exercise associated hyponatremia is a practical concern for the event or training session. In large team settings, it may be more feasible or efficacious to focus efforts on athletes that consistently report to practice with considerable loss in body mass between practices or repeatedly manifest other markers (e.g., elevated urine specific gravity values) indicative of chronic pre-exercise hypohydration. There is no empirical evidence that individuals will alter their fluid intake strategies upon recognition of accurate sweat loss information. It is also apparent that study participants were not basing their fluid intake during or between training bouts on the volume of sweat they believe they are losing during training. Current findings should be interpreted with these considerations in mind, and also recognize that there is a dearth of data concerning professional athletes. The authors hope these findings will be considered in development of future hydration guidelines.

## Figures and Tables

**Table 1:** Synopsis of sweat loss estimation investigations.

Study	Participants	Exercise Condition	Sweat Loss
	Sex = *n*	Modality: Duration (M ± SD; Range)	Actual (L)
	Age (Years)	Environmental Conditions (M ± SD; Range)	Predicted (L); Prediction Method
	Training Status	Sweat Loss as % of Body Mass	% of Actual
Campbell et al. (2017) [15]	F = 18 M = 3 33 ± 11 Recreational	Hot Hatha yoga; 60 min Dry = 38.7 ± 2.7 °C Relative humidity = 35.8 ± 13.3% 1.11 ± 0.57%	0.814 ± 0.539 0.512 ± 0.335; 1000 mL bottles 72.5 ± 49.0%
Cronin et al. (2016) [14]	F = 20 M = 30 30 ± 9 Recreational	CrossFit^™^; 34.3 ± 5.5 min WBGT = 20.1 ± 2.8; 16.0–22.7 °C 0.91 ± 0.31%	0.746 ± 0.305 0.655 ± 0.404; 250 mL bottles 96.0 ± 60.8%
Davis et al. (2019) [18] ^A^	M = 12 22 ± 2 Recreational	Treadmill running-temperate environment; 60 min WBGT = 18.1 ± 0.2 °C 1.72 ± 0.29%	1.348 ± 0.282 0.793 ± 0.333; paper race cups 60.5 ± 26.6%
Davis et al. (2019) [18] ^B^	M = 12 22 ± 2 Recreational	Treadmill running-hot environment; 60 min WBGT = 25.6 ± 0.5 °C 2.43 ± 0.56%	1.907 ± 0.529 1.198 ± 0.550; paper race cups 66.7 ± 35.4%
O’Neal et al. (2012) [10]	F = 20 M = 19 41 ± 11 Recreational to collegiate	Outdoor running: 59.14 ± 3.46 min WBGT = 24.1 ± 1.5; 21.3–27.7 °C 2.11 ± 0.52%	1.468 ± 0.484 0.738 ± 0.470; paper race cups 50.4 ± 23.0%
Shaver et al. (2018) [11]	F = 23 (2 trials) 26 ± 6 Recreational	Outdoor running 15-km time trial; 79.98 ± 7.00 min WBGT = 20.0 ± 2.8; 12.8–25.6 °C 2.20 ± 0.48%	1.306 ± 0.307 0.498 ± 0.516; water bottle reference 39.9 ± 40.9%
O’Neal et al. (2014) [13]	F = 8 M = 12 20 ± 2 Recreational to collegiate	Outdoor running; 59.91 ± 3.42 min WBGT = 20.1 ± 2.8; 16.0–22.7 °C 1.96 ± 0.51%	1.374 ± 0.423 0.697 ± 0.600; paper race cups 50.1 ± 39.1%
Thigpen et al. (2014) [12] ^A^	F = 11 M = 11 20 ± 1 NCAA Division II	Collegiate basketball conditioning practice; (F = 95 min, M = 46 min) WBGT F = 20.4, M = 20.0 °C F = 1.47 ± 0.27, M = 1.13 ± 0.27%	F = 1.112 ± 0.271 M = 0.969 ± 0.250 F = 0.394 ± 0.242 M = 1.316 ± 0.847; standard practice water bottle F = 37.5 ± 28.3 M = 142.6 ± 102.4%
Thigpen et al. (2014) [12] ^B^	F = 11 M = 11 20 ± 1 NCAA Division II	Collegiate basketball in-season practice; (F = 170 min, M = 170 min) WBGT F = 18.5; M = 17.2 °C F = 2.53 ± 0.43, M = 2.90 ± 0.50%	F = 1.910 ± 0.441 M = 2.471 ± 0.495 F = 0.632 ± 0.284 M = 1.740 ± 1.201; standard practice water bottle F = 35.1 ± 20.1 M = 70.6 ± 56.9%
Muth et al. (2019) [16] ^A^	F = 16 20 ± 1 NCAA Rugby Union	Outdoor rugby practice; 95 min Dry = 11.0 ± 1.4 °C Relative humidity = 57.5 ± 3.5% 1.19 ± 0.55%	0.928 ± 0.446 0.335 ± 0.243; 532 mL cup 53.0 ± 69.2%
Muth et al. (2019) [16] ^B^	M = 20 20 ± 1 NCAA Rugby Union	Outdoor rugby practice; 90 min Dry = 8.5.0 ± 2.1 °C Relative humidity = 46.5 ± 9.2% 2.00 ± 0.54%	1.917 ± 0.516 0.373 ± 0.183; 532 mL cup 20.9 ± 12.5%
Love et al. (2018) [17]	M = 20 25 ± 4 Professional	Outdoor rugby practice; 120 min Dry = 35.0 °C Relative humidity = 40% 3.2 ± 0.9%	3.291 ± 0.948 0.898 ± 0.575; 532 questionnaire 27.4 ± 17.2%

Abbreviations: F = Female, M = Male, NCAA = National Collegiate Athletic Association, WBGT = Wet Bulb Global Temperature. ^A, B^ = sub sets of data from same study.

**Table 2:** Cumulative comparisons of sweat loss estimation variables for all participants and by sex (mean ± SD).

	Female (*n* = 147 *)	Male (*n* = 150 **)	All (*n* = 297)
Age (years)	27.3 ± 9.7	26.4 ± 9.6	26.8 ± 9.7
Weight (kg)	66.2 ± 11.1	87.1 ± 13.5†	76.7 ± 16.2
Exercise duration (min)	77 ± 33	74 ± 38	75 ± 36
Sweat loss			
% body mass	1.64 ± 0.71	2.04 ± 0.92 †	1.84 ± 0.84
Absolute (L)	1.072 ± 0.473 ‡	1.778 ± 0.907 †‡	1.428 ± 0.806 ‡
Estimated (L)	0.481 ± 0.372	0.908 ± 0.666 †	0.697 ± 0.581
Accuracy (%)	55.2 ± 51.5	62.4 ± 54.5	58.9 ± 53.1

* = 147 estimations were made by 116 female participants. ** = 150 estimations were made by 127 male participants. † = *p* < 0.001 for comparison between sexes. ‡ = *p* < 0.001 for comparison made between estimated and actual sweat loss volume.

**Table 3:** Contingency table for sweat loss estimation accuracy comparisons (values = *n* (% of total sample)).

	**Absolute Volume Comparisons**			
Estimated Sweat Loss (L)	Actual Sweat Loss (L)			
	0–1	1–2	2–3	3+
0–1	82 (27)	122 (41)	29 (9)	12 (4)
1–2	7 (2)	22 (7)	9 (3)	5 (1)
2–3	1 (0.3)	3 (1)	4 (1)	0 (0)
3+	0 (0)	0 (0)	1 (0.3)	0 (0)
	**Relative comparisons**			
Estimation accuracy (%)	Sweat loss as a % of body mass			
	0–1	1–2	2–3	3+
0–50	17 (5)	74 (24)	55 (18)	22 (7)
50–100	16 (5)	33 (11)	21 (7)	7 (2)
100–150	10 (3)	17 (5)	4 (1)	3 (1)
150+	12 (4)	4 (1)	2 (0.6)	0 (0)
